# A combined electrophysiological and morphological examination of episodic memory decline in amnestic mild cognitive impairment

**DOI:** 10.3389/fnagi.2013.00051

**Published:** 2013-09-23

**Authors:** Michael Hoppstädter, Andrea Victoria King, Lutz Frölich, Michèle Wessa, Herta Flor, Patric Meyer

**Affiliations:** ^1^Department of Cognitive and Clinical Neuroscience, Central Institute of Mental Health, Medical Faculty Mannheim, Heidelberg UniversityMannheim, Germany; ^2^Division of Gerontopsychiatry, Central Institute of Mental Health, Medical Faculty Mannheim, Heidelberg UniversityMannheim, Germany; ^3^Department of Clinical Psychology and Neuropsychology, Mainz UniversityMainz, Germany

**Keywords:** event-related potentials, familiarity, medial temporal lobe, recognition memory, voxel-based morphometry

## Abstract

Early stages of Alzheimer’s disease (AD) are characterized by neuropathological changes within the medial temporal lobe cortex (MTLC), which lead to characteristic impairments in episodic memory, i.e., amnestic mild cognitive impairment (aMCI). Here, we tested the neural correlates of this memory impairment using event-related potentials (ERPs) and voxel-based morphometry. Twenty-four participants were instructed to encode lists of words and were tested in a yes/no recognition memory task. The dual-process model of recognition memory dissociates between acontextual familiarity and recollection of contextual details. The early frontal ERP old/new effect, which is thought to represent a neural correlate of familiarity-based memory, was absent in aMCI, whereas the control group showed a significant early old/new effect at frontal electrodes. This effect was positively correlated with behavioral episodic memory performance. Analyses of brain morphology revealed a focused gray matter loss in the inferior and medial temporal lobes in aMCI versus healthy controls. Moreover, the positive correlation between gray matter volume in the MTLC and the familiarity-related early frontal old/new effect supports the notion that this effect relies upon the integrity of the MTLC. Thus, the present findings might provide a further functional marker for prodromal AD.

## INTRODUCTION

Recently, the idea of a transitional phase from healthy aging to Alzheimer’s disease (AD) was increasingly investigated. This precursory stage has mostly been described as mild cognitive impairment (MCI). As early AD symptomatology is most prominently related to deficiencies in episodic memory function, those are of special interest for an early diagnosis of AD and also for the definition of MCI as a putative AD prodrome. Because the AD-related neuropathology begins in the perirhinal cortex (Braak and Braak, 1991; Delacourte et al., 1999), an examination of the cognitive functions relying on this cortical region in MCI patients might further substantiate cognitive changes during the early pathologic stages of AD.

Numerous findings from behavioral, electrophysiological and imaging studies argued for a dual-process model of recognition memory (Yonelinas, 2002), dissociating an acontextual feeling of knowing (familiarity) that something has occurred before from the conscious retrieval of contextual details (recollection). Recent neuroanatomical models assume a functional dissociation within the medial temporal lobe (MTL) with regard to recollection and familiarity. Data from patients suffering from MTL lesions (e.g., Aggleton and Brown, 1999; Bowles et al., 2007) and functional imaging data (for an overview, see Eichenbaum et al., 2007) indicate that a recollection deficit is associated with damage to the hippocampus while lesions to the surrounding medial temporal lobe cortex (MTLC; i.e., the perirhinal and entorhinal cortices) trigger a familiarity deficit.

Familiarity and recollection have been differentiated electrophysiologically using event-related potentials (ERPs). An early old/new effect (hits – correct rejections; Rugg et al., 1998), which peaks at frontal electrode sites at about 400 ms after stimulus onset, has been related to familiarity processing and can be dissociated from a later effect emerging at about 500 ms over the left parietal scalp that is associated with recollection-based processing (Friedman et al., 2010). At least for recollection, this functional mapping onto divergent MTL structures has been corroborated by studies that showed the absence of the late parietal ERP effect when hippocampal tissue was damaged (Düzel et al., 2001; Addante et al., 2012). For familiarity, Bowles et al. (2007) reported the lack of a behavioral correlate in a patient with a lesion sharply restricted to the MTLC, leaving hippocampal tissue intact. Effects similar to the ERP early old/new effect have been described in the MTLC with intracranial recordings (Grunwald et al., 1998; Fernandez and Tendolkar, 2006) and with magnetoencephalography (Düzel et al., 2003; Gonsalves et al., 2005). However, the link between the MTLC and the scalp-recorded electrophysiological correlate of familiarity is so far missing.

From a developmental perspective, a large body of literature reports concordantly that in comparison to younger adults memory performance in older adults is associated with an impairment of recollection while familiarity seems to be unaffected or even boosted (e.g., Yonelinas, 2002; Dennis and Cabeza, 2008). In line with this observation, structural imaging results show that the hippocampus, constituting the crucial structure for recollection (Eichenbaum et al., 2007), is more severely affected by age-related volume loss than the MTLC (Raz et al., 2005), which has been related to familiarity-based processing.

Electrophysiological investigations of the differential vulnerability of recognition memory processes in aging found an exclusive reduction of the recollection-related ERP effect. Friedman et al. (2010) reported a frontal old/new effect from adolescence onwards and a parietal old/new effect across age groups but drastically reduced in old age. However, it has been shown that very high-performing older adults still display a preserved parietal old/new effect indicating that recollection might be intact in certain older subpopulations (Duarte et al., 2006; Wang et al., 2011).

When this age-related cognitive decline in aging is superimposed by AD, cognitive abilities may gradually deteriorate because of the progressive nature of neuropathological changes in AD. In the prodromal phase of AD, neuropathological changes have already evolved to a point where specific mild cognitive symptoms are found, however, they may not be severe enough to be diagnosed as dementia. This transitional phase has been described phenomenologically as MCI. Different MCI subtypes have been differentiated depending on the extent of cognitive malfunctions, meaning if only one or several cognitive domains are compromised (Petersen, 2004). A singular memory impairment is characterized as single-domain amnestic MCI (aMCI). If multiple cognitive domains are impaired, this is classified as multiple-domain MCI (mdMCI). In comparison to aMCI, this subtype might not constitute a prodromal phase specific to the development of AD (Petersen, 2004). If MCI is accompanied by typical changes of biomarkers for AD, the terms “MCI due to AD” or “prodromal AD” have been proposed (Dubois et al., 2010; Sperling et al., 2011).

The volume of MTL structures in AD and MCI was for example studied using tracing methods or voxel-based morphometry (VBM) with scans from structural magnetic resonance imaging (MRI). AD patients showed significant neuronal atrophy in the MTL including the hippocampus (e.g., Karas et al., 2004; Barbeau et al., 2008; Pihlajamäki et al., 2009; Schmidt-Wilcke et al., 2009), whereas MCI subjects displayed significant neuronal loss only in the MTLC (Kordower et al., 2001). Regionally specific atrophy within the MTL also correlated differentially with functionally distinct memory measures, e.g., delayed recognition with MTLC volume but delayed free recall with hippocampal size in a mild AD population (Wolk and Dickerson, 2011). Also using multiple behavioral recognition assessments, hippocampal volume was more strongly related to recollection and MTLC volume more strongly related to familiarity estimates (Wolk et al., 2011). Structural MRI data suggest that the differentiation of MCI subtypes based on cognitive criteria also holds for the underlying atrophy processes with more focused MTL atrophy in aMCI subjects, and more diffuse atrophy in mdMCI (Bell-McGinty et al., 2005).

The hypothesis that pronounced MTLC atrophy in aMCI leads to familiarity impairments yielded conflicting results in previous behavioral studies. Most studies investigating recollection-based memory indeed found significantly reduced recollection estimates and some of the studies found an additional deficit of familiarity estimates (e.g., Wolk et al., 2008; Ally et al., 2009; Algarabel et al., 2009, 2012) However, others reported no significant differences for familiarity measures between controls and MCI subjects (e.g., Westerberg et al., 2006; Anderson et al., 2008; Hudon et al., 2009; Serra et al., 2010). However, it might be a substantial confound that most of the studies investigated mdMCI subjects though atrophy in mdMCI is not restricted to the MTLC (Petersen, 2004; Whitwell et al., 2007).

Recently, Ally et al. (2009) investigated ERPs to verbal and visual recognition memory in healthy older adults and in a mixed group of single and multiple domain aMCI. The aMCI group showed a significantly worse performance than the control group across conditions. ERP results revealed a recollection-based parietal old/new effect for controls in the picture but not in the word condition, whereas aMCI subjects did not show any recollection correlate in either task. A frontal old/new effect that indicates familiarity-based processing was similarly present in both groups in the picture condition wheras in the word task only controls showed a significant frontal old/new effect. This suggests that aMCI patients exhibit deficient familiarity and recollection-based processing in recognition memory tasks involving word material.

To investigate more thoroughly the impact of aMCI on electrophysiological markers of recognition memory processes, a verbal yes/no recognition memory paradigm was designed to analyze alterations of the frontal and parietal ERP old/new effects indicating recollection and familiarity, respectively, in a sample of single-domain aMCI subjects. Given that also healthy older adults potentially exhibit deficient recollection (Friedman et al., 2010) there was no specific hypothesis on the parietal old/new effect. However, the frontal old/new effect was hypothesized to differ between aMCI patients and control subjects indicating differential reliance on familiarity. Moreover, these group differences should correspond to morphological changes in the MTL assessed in a VBM analysis with a reduced volume of MTLC gray matter in aMCI subjects.

## MATERIALS AND METHODS

### PARTICIPANTS

Fourteen individuals (mean age 67.8 years, four female) who met criteria for single-domain aMCI (Petersen et al., 1999; Winblad et al., 2004) formed the patient group and ten healthy and cognitively intact individuals acted as a control group (mean age 68.0 years, six female). Both groups were matched for age and level of education. **Table 1** provides demographic characteristics and performance on neuropsychological tests of the two groups. Subjects with aMCI were recruited from the Memory Clinic of the Central Institute of Mental Health or by invitations sent by mail to a random sample of older residents in the Mannheim region (all healthy controls). All subjects gave written informed consent prior to study start. The study was approved by the ethics committee of the Medical Faculty Mannheim, University of Heidelberg and was conducted in accordance with the Declaration of Helsinki. For the VBM analyses, one aMCI subject had to be excluded due to metallic implants.

**Table 1:** Measures of clinical and global functioning, episodic memory, and executive functioning and statistical comparison of the patient and control group (N or mean value, SD in parentheses).

	Patients	Controls
Number of subjects	14	10
Mean age (years)	68.00 (4.00)	67.80 (4.69)
Female/male	4/10	6/4
Mean years of education (years)	11.30 (2.50)	12.90 (3.80)
**Assessment of clinical/global functioning**
Hamilton rating scale for depression (HAMD)	0.50 (0.94)	0.89 (1.05)
General depression scale (ADS-L)	4.82 (2.60)	7.00 (6.86)
State-trait anxiety inventory-trait (STAI-T)	33.29 (9.28)	27.78 (7.93)
Competence rating scale (MKS, self-report): functional abilities	54.08 (5.49)	59.13 (1.64)
Competence rating scale (MKS, self-report): cognitive abilities	45.15 (8.12)	54.25 (3.69)
Competence rating scale (MKS, relative’s form): functional abilities	56.27 (4.40)	59.71 (0.75)
Competence rating scale (MKS, relative’s form): cognitive abilities	49.20 (8.12)	57.14 (4.25)
Mehrfachwahl- Wortschatz-Intelligenztest - B (IQ-score)	100.69 (11.54)	109.25 (11.65)
CANTAB simple reaction time: mean correct latency	270.48 (65.8)	244.87 (21.84)
CERAD: Boston Naming Test	14.66 (0.92)	14.35 (0.70)
CERAD: MMSE	27.85 (1.29)	28.88 (1.05)
**Assessment of mnemonic functioning**
CERAD: word list memory test: total trials 1–3	20.57 (4.44)	24.11 (2.89)
CERAD: word list memory test: delayed recall	5.92 (1.81)	8.77 (0.97) ^a^
CERAD: word list memory test: delayed recall savings (%)	72.57 (18.13)	94.04 (5.70)^a^
CERAD: word list memory test: recall intrusions	1.21 (1.71)	0.22 (0.44)
Logical memory immediate (WMS-R)	22.78 (5.92)	29.33 (3.67)
Logical memory delayed (WMS-R)	18.35 (7.03)	26.00 (2.95) ^a^
CANTAB delayed matching to sample test: percent correct (all delays)	76.66 (9.71)	84.99 (5.63)
CANTAB paired associates learning test: total errors (adjusted)	32.92 (18.58)	12.00 (7.11) ^a^
**Assessment of executive functioning**
CANTAB intra-extra dimensional set shift test: total errors (stage 9)	4.46 (7.03)	2.10 (3.63)
CANTAB intra-extra dimensional set shift test: total errors (stage 7)	1.30 (0.63)	1.00 (0.00)
CANTAB intra-extra dimensional set shift test: total errors (stage 5)	2.23 (2.86)	1.60 (1.57)
CANTAB intra-extra dimensional set shift test: total errors (stage 2)	1.61 (0.96)	1.6 (0.69)
CANTAB spatial span forward	4.92 (0.73)	5.5 (0.52)
CANTAB spatial span reverse	4.71 (0.82)	5.40 (0.84)
CERAD: trail making test A	48.57 (12.31)	37.88 (10.45)
CERAD: trail making test B/A	2.18 (0.49)	2.00 (0.36)
CERAD: verbal fluency phonological	12.78 (3.66)	15.77 (4.65)

*CANTAB, Cambridge Neuropsychological Test Automated Battery; CERAD, Consortium to Establish a Registry for Alzheimer’s Disease; MKS, Marburger Kompetenz-Skala; MMSE, Mini Mental State Examination; WMS-R, Wechsler Memory Scale-Revised.*

a *P*< 0.05 in Bonferroni-adjusted *t*-tests between aMCI and control subjects.

### CLINICAL ASSESSMENT

All participants were between sixty and seventy-five years of age and German native speakers. Individuals interested in the study participated in an initial telephone screening addressing demographic details, physical status, exclusion criteria and general cognitive status assessed by the modified Telephone Interview of Cognitive Status (TICS-M; Brandt et al., 1988). All patients were diagnosed after clinical history, medical and neurological examination, scoring on the Mini Mental State Examination (MMSE), and after neuropsychological assessment with neuropsychological tests assessing attention, psychomotor speed, verbal fluency, orientation, executive functions, constructional praxis, and episodic memory taken from the Consortium to Establish a Registry for Alzheimer’s Disease (CERAD-Plus; Morris et al., 1989), the Cambridge Neuropsychological Test Automated Battery (CANTAB, Cambridge Cognition, Cambridge, UK) and the Wechsler Memory Scale – Revised (WMS-R; Wechsler, 1987). All participants were screened for mental disorders by the Structured Clinical Interview for DSM-IV (SCID I; First et al., 2002). For an overview of clinical and neuropsychological tests, see **Table 1**.

Additionally, the patient and relative’s form of the Marbuger Kompetenz-Skala (MKS; Gauggel et al., 1998) were used as external measures for ratings of impairment of daily functioning. In addition, subjects underwent a structural MRI examination and images were screened for probable exclusion criteria by an experienced neuroradiologist before subjects were included in the study.

To ensure the assignment to the patient group, differences of individual test scores between the patient and the control group were computed with Bonferroni-adjusted *t*-tests to account for neuropsychological deficits of aMCI patients in the memory domain. Participants were diagnosed as having aMCI if they fulfilled the following criteria (Petersen et al., 1999; Winblad et al., 2004): (1) concerns regarding memory decline, corroborated by a patient’s relative, (2) objective memory impairment defined by performance at or lower than 1.3 standard deviations below the mean value (i.e., under the tenth percentile) of an age- and education-matched norm population on test indices of one or more of the employed memory tests (see **Table 1**), (3) preserved general cognitive functioning defined by performance at least above 1.3 standard deviations below the mean on all other measures not assessing memory, (4) independence of functioning in daily life as assessed with the MKS, (5) not demented or suffering from conditions that may cause memory deficits as evaluated by medical history, MRI examination and structured clinical interviews.

All participants were required to have a negative history for medical disorders (e.g., diabetes, untreated vitamin deficiencies, disorders of the thyroid, anemia, sleep apnea, other significant concurrent physical illnesses), neurological brain diseases [e.g., stroke, cerebral neoplasm, hemorrhage, inflammation, Parkinson’s disease, vascular encephalopathy with increased white matter (WM) lesions], and mental disorders (e.g., affective disorders). Additionally, all subjects had to have normal or corrected to normal visual acuity and contrast sensitivity and had to be free of metallic biomedical devices (for MRI scan). Current drug intake of dopaminergic or serotonergic agents, beta-adrenergic blockers or benzodiazepines was ruled out.

### PROCEDURE AND EXPERIMENTAL PARADIGM

Subjects were seated in a high-back armchair in a sound-dampened and dimly illuminated chamber at a distance of 100 cm from a 17-inch monitor. All stimuli were displayed in white against a black background in Courier New 25 point font. All experimental details were displayed in central vision on the screen. The two experimental phases were separated by a short self-paced recreational break. The experiment was designed using E-Prime software (Psychology Software Tools, Pittsburgh, PA, USA). An initial learning phase was followed by a test phase. To facilitate the task for the subjects and to ensure above chance performance, the study list was repeated after initial presentation thus leading to two identical consecutive study phases. For additional facilitation, the recognition paradigm was split into two runs with both parts again subdivided into two identical study phases and one test phase. The two runs were of equal construction and size, but contained different sets of stimuli. Overall 200 concrete German nouns of moderate frequency (5–300 occurrences per million) and length (4–10 letters) were used. Frequency was determined using the German language database of the Dutch Centre for Lexical Information (CELEX; Baayen et al., 1993).

Within a run, one study phase consisted of 50 single word stimuli. To make sure that subjects processed and encoded the words, they had to do a categorical subjective pleasantness rating and judge each word as either positive or negative using two different keys. Additionally, they were instructed to carefully examine the words because of a later recognition test. Each trial started with a fixation cross, which was displayed for 500 ms in order to minimize eye movements and guide the subject’s attention to the location where the target stimulus appeared. The target was preceded by a blank screen for 300 ms and followed by another 50 ms blank screen whereby the word stimulus itself was displayed for 2000 ms. The trial ended with a screen indicating “positive/negative?” lasting for 1500 ms, which also was the response window for the subjects’ pleasantness judgment. The test phase was appended after the repetition of the study phase and consisted of the 50 studied words and 50 new words. A 500 ms fixation cross and a 300 ms blank screen were followed by the target stimulus, which was displayed for 2000 ms and faded to a 50 ms blank screen. Accordingly, a 2000 ms response window, which indicated the question “old or new?,” requested the subjects to make their recognition judgment by button press. The item order of the test lists was pseudo-randomized, so that the same item type (old or new) was not presented consecutively more than three times. Moreover, there were two versions of stimulation lists (A and B) for the study and test phases, which were counterbalanced across subjects. Subjects who studied list A, saw the items of list B as new items in the test phase, and vice versa.

### ERP RECORDING AND ANALYSIS

The continuous electroencephalogram (EEG) was recorded using BrainVision Recorder (Brain Products, München, Germany) with an Easy Cap (EASYCAP, Herrsching, Germany) at 64 equidistant Ag/AgCl scalp electrodes. All electrodes were recorded with reference to the left mastoid, and were later re-referenced offline to the average of left and right mastoid. Additionally, vertical and horizontal eye movements were recorded from bipolar electrodes above and below the right eye and on the outer canthi of both eyes. All channels were amplified with a pass band from DC to 1000 Hz and a resolution of 0.1 μ V and were digitized at a sampling rate of 250 Hz. Electrode impedances were kept below 5 kΩ.

Offline data processing was performed with BrainVision Analyzer (Brain Products, München, Germany) and EEProbe (ANT Software, Enschede, The Netherlands). First, an artifact correction based on an independent component analysis was conducted where factors representing EOG artifacts such as blinks, horizontal eye movements or pulse artifacts were eliminated. The further data processing comprised a digital band-pass filter set to 0.1 and 30 Hz. The continuous EEG was separated into epochs of 1500 ms, including a 300 ms baseline. Epochs which still contained EOG or other artifacts were rejected by visual inspection before averaging.

### IMAGE ACQUISITION AND ANALYSIS

Magnetic resonance imaging data were acquired from a 3 Tesla magnetic resonance scanner (Magnetom Trio, Siemens Medical Solutions, Erlangen, Germany). For each subject, a T1-weighted gradient echo MP-RAGE (magnetization prepared rapid gradient echo) sequence (repetition time = 2300 ms, echo time = 2.98 ms, flip angle 9°, field-of-view = 256 × 256 mm, voxel size = 1.0 × 1.0 × 1.1 mm, 160 sagittal slices) was recorded.

To identify significant regional differences between the control and the aMCI group and to analyze correlations between ERP effects and gray matter (GM) values, VBM was applied. In contrast to manual tracing methods, VBM permits to investigate the presence of between-group differences in GM volumes across the whole brain without a priori decisions about which structures to evaluate. Moreover, VBM is an automated, rater-independent method, and provides highly reproducible results (Busatto et al., 2008). Data pre-processing and analysis were performed using SPM8^5^ (Wellcome Department of Imaging Neuroscience, London, UK). Pre-processing of the data involved visual inspection of the T1-weighted images to control for imaging artifacts and the consecutive segmentation into GM, WM and cerebrospinal fluid (CSF), building a template for GM out of 21 images of healthy seniors (containing the ten control subjects of the EEG study plus an additional eleven subjects of a larger sample without EEG data) through an iteratively non-linear registration algorithm (DARTEL Toolbox for SPM8; Good et al., 2001; Ashburner, 2007) and a normalization of this template to the Montreal Neurological Institute template^6^. The Jacobian determinants derived from the normalization procedure were used to obtain modulated VBM data which allow for the comparison of regional volume differences. Individual GM images were smoothed with an isotropic Gaussian kernel of 6 mm full-width at half-maximum before entering them into statistical analysis. Global volumes of GM, WM, and CSF were estimated from segmented images using the VBM8 toolbox for SPM8^7^ and summed to generate an estimate for total intracranial volume (TIV).

### STATISTICAL ANALYSIS

All analyses were carried out on the EEG data of the test phases of the two separate runs which were concatenated prior to EEG preprocessing. For all calculations of ERP effects, the mean amplitudes of two previously specified time windows were extracted and were contrasted between defined regions of interest (ROIs), each containing six electrodes, by averaging the mean amplitudes of these six electrode sites to a compound measure. The time windows were 450–600 ms after stimulus onset (early time window) and 600–800 ms after stimulus onset (late time window). The early time window was analyzed in two frontal ROIs: anterior left hemisphere (F1, F3, F5, FC1, FC3, FC5) and anterior right hemisphere (F2, F4, F6, FC2, FC4, FC6). The late time window was investigated in two corresponding parietal ROIs: posterior left hemisphere (CP1, CP3, CP5, P1, P3, P5) and posterior right hemisphere (CP2, CP4, CP6, P2, P4, P6). Statistical comparisons were computed using multivariate analyses of variance (MANOVAs) as recommended by Dien and Santuzzi (2005). All statistics for the ERP effects were carried out in SPSS, version 18 (SPSS Inc., Chicago, IL, USA).

To assess the relationship between the early frontal old/new effect reflecting familiarity and the mnemonic performance level of the participants, Pearson correlations were computed. The ERP effect was quantified in the early time window for ROIs with significant differences between the task-relevant item types by subtracting the mean amplitude of new items from the corresponding mean amplitude of old items. Episodic memory performance was quantified by two values. First, the performance in the current task was standardized by *z*-transforming individual task performance, thus establishing the possibility to directly relate behavioral performance to associated electrophysiological measures. Secondly, episodic memory performance was quantified using data of the neuropsychological testing. Therefore, the eight test scores of episodic memory (see **Table 1**) were averaged to a compound score (original scores were first *z*-transformed).

In order to obtain a standardized value of recognition performance, percentile ranks (Pr scores) were calculated. The Pr is calculated from the probability of hits (i.e., a correct old response) minus the probability of false alarms (FA; i.e., a wrong old response), whereby hit and FA rates were corrected as suggested by Snodgrass and Corwin (1988) to avoid rates of 0 and 1. Also the response bias (Br score) was quantified via the formula Br = FA/(1 - Pr), resulting in values smaller than 0.5 for a more conservative response bias and larger than 0.5 for a more liberal response bias (Snodgrass and Corwin, 1988). Statistics for behavioral measurements of the recognition memory paradigm were performed using two-tailed *t*-tests.

Global volume differences in GM, WM, and CSF were calculated with a univariate analysis of variance. Because GM volume changes due to beginning AD-related neuropathology were in the focus of this investigation, only GM segments were subjected to VBM analysis. A cohort analysis (two sample *t*-test) with age, gender and TIV as nuisance variables was performed and, in addition, a regression analysis across both groups was computed to identify brain regions that showed a significant correlation between GM values and the quantified ERP effects, again accounting for age, gender and TIV. However, due to the propensity of structural MRI scans to show susceptibility artifacts in the MTL, we allowed a less conservative statistical threshold (*P* < 0.005, uncorrected for multiple comparisons) for analyses within this region, whereas clusters outside of the MTL are reported uncorrected at *P* < 0.001. Additionally a cluster extent of 40 contiguous voxels was included into both VBM analyses.

## RESULTS

### NEUROPSYCHOLOGICAL EXAMINATION

The patient group performed significantly poorer than the control group in four of the critical measures of episodic memory functioning. Moreover, there were no significant between-group differences for measures of global and clinical functioning or for the tests of executive function or other non-memory tests (e.g., Boston Naming Test, verbal fluency, constructive praxis).

### BEHAVIORAL RESULTS

**Table 2** lists Pr scores, reaction times and response bias for the patient and control group. For both, Pr scores [*t*(22) = -3.56, *P* < 0.01] and reaction times [*t*(22) = 2.65, *P* < 0.05], there was a significant between-group difference indicating that the aMCI patients performed worse than the control subjects and were slower. There was no significant between-group difference for response bias [*t*(22) = -4.84, *P* = 0.63], which deviated only marginally from 0.50 in both groups, thus denoting that neither the patients nor the control subjects had a tendency to a more liberal or conservative response behavior.

**Table 2:** Mean performance (Pr) and response bias (Br) scores and reaction times of patient and control group in the recognition memory task (SD in parentheses).

	Patients	Controls
Pr	0.74 (0.12)	0.90 (0.06)^a^
Br	0.43 (0.23)	0.48 (0.23)
Reaction time (ms)	1670.58 (240.16)	1351.37 (351.94)^b^

*All values differ significantly from zero.*

a *P*< 0.01

b *P*< 0.05

### MORPHOLOGICAL ANALYSES

**FIGURE 1:**
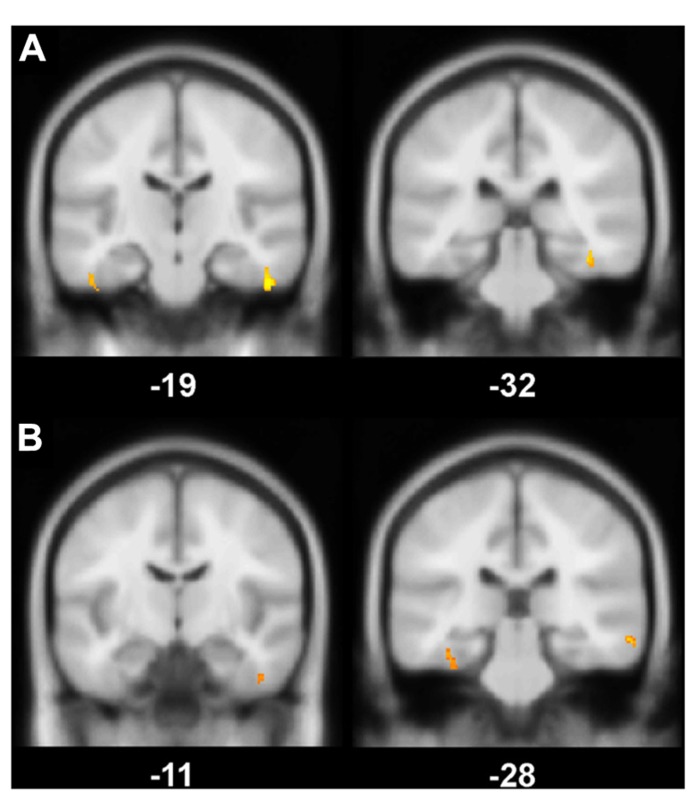
**(A)** Gray matter (GM) volume differences in patients compared to controls (contrast controls > patients). The slice on the left shows volume reductions in left and right fusiform gyrus (BA 20), the slice on the right illustrates volume loss in the right perirhinal cortex (BA 36); **(B)** Positive correlation between the magnitude of the familiarity old/new effect with GM volume. On the left, a cluster in right fusiform gyrus (BA 20) correlates significantly with the familiarity effect, on the right, there is a significant correlation between left perirhinal cortex (BA 36) and the familiarity effect; Significance level is *P*_uncorrected_ < 0.005 with a cluster threshold of 40 contiguous voxels.

Global GM, WM, and CSF volumes between the aMCI and the control group revealed significant volume differences only for CSF [*F*(1,21) = 5.15, *P* < 0.05] with a greater CSF volume in aMCI patients, but neither for GM, WM nor for TIV (all *P* > 0.1; see **Table 3**). Second, a two sample *t*-test between the patient and the control group showed that GM loss in the aMCI group was focused bilaterally in the inferior and medial temporal lobes (see **Figure 1A** and **Table 4**).

**Table 3:** Global volumes (in ml) of brain tissues and fluids.

Parameter	Patients	Controls
Gray matter volume	614.37 (40.44)	605.10 (38.46)
White matter volume	512.13 (36.25)	496.19 (47.61)
CSF volume	289.75 (46.62)	250.27 (33.14)^a^
TIV	1416.25 (99.11)	1351.56 (86.29)

*CSF, cerebrospinal fluid; TIV, total intracranial volume.*

a *P*< 0.05

**Table 4:** (a) Regional gray matter differences between controls and patients and (b) regions where gray matter volume positively correlates with the magnitude of the frontal old/new effect.

Region		Brodmann area	Talairach coordinates [mm] of peak activation			cluster size	*P*-level_**uncorrected**_	Peak *T* value
		*x*	*y*	*z*	*k*
**(a) Two sample *t*-test: controls>patients**
Fusiform gyrus	R	BA 20	48	-22	-25	124	<0.005	4.05
Perirhinal cortex	R	BA 36	37	-28	-11	47	<0.005	4.01
Inferior temporal gyrus	L	BA 20	-45	-18	-19	49	<0.005	3.73
			-41	-23	-28		<0.005	3.32
Middle temporal gyrus	R	BA 21	50	0	-21	47	<0.005	3.70
Lingual gyrus	R	BA 18	15	-73	-5	95	<0.001	5.80
			12	-79	1		<0.001	4.84
**(b) Regression analysis with ERP familiarity effect as predictor: positive correlations**
Middle temporal gyrus	R	BA 21	56	-32	-4	437	<0.005	4.99
		62	-38	-13		<0.005	6.81
Fusiform gyrus	R	BA 37	51	-44	-9		<0.005	5.80
Middle temporal gyrus	R	BA 21	49	3	-30	85	<0.005	4.53
Inferior temporal gyrus	R	BA 20	49	-7	-30		<0.005	2.96
Perirhinal cortex	L	BA 36	-34	-29	-17	59	<0.005	3.84
Fusiform gyrus	R	BA 20	43	-10	-26	40	<0.005	3.83
Lingual gyrus	L	BA 19	-12	-54	0	82	<0.001	5.27
Posterior cingulate gyrus	L	BA 30	-21	-51	1		<0.001	4.57
Insula	R	BA 13	39	2	1	109	<0.001	5.15
Dorsolateral prefrontal gyrus	L	BA 46	-45	40	9	64	<0.001	4.88
			-42	32	7		<0.001	4.85
Ventral anterior cingulate gyrus	L	BA 24	-5	2	35	49	<0.001	4.52
Insula	L	BA 13	-38	7	-3	43	<0.001	4.35

* 
All *P*-values include a cluster threshold of 40 contiguous voxels.*

*BA, Brodmann area; ERP, event-related potential; L, left hemisphere; R, right hemisphere; TIV, total intracranial volume; *x*, *y* and *z* coordinates according to the atlas of Talairach and Tournoux, coordinates mark the location of highest T value within a corresponding cluster.*

### ERP RESULTS

**FIGURE 2:**
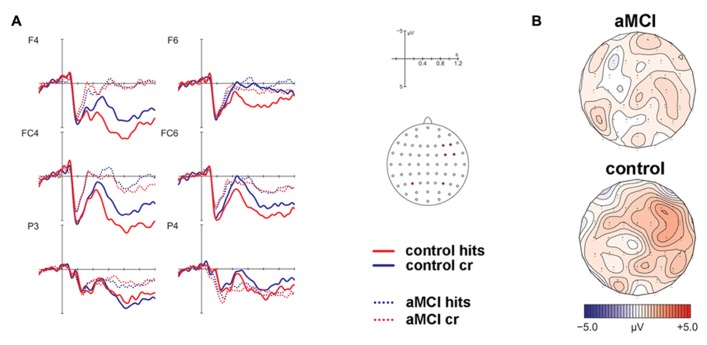
**Results of the event-related potential analysis of the right anterior region of interest (electrodes F4, F6, FC4, FC6; not displayed are F2, FC2).** Waveforms are shown in panel **(A)**. Solid lines indicate the ERPs of controls, dotted lines mark ERPs of patients. Hits are given in red color, correct rejections (cr) in blue color. At posterior electrodes (exemplified at positions P3/P4), there was no significant effect for hits vs. cr. The corresponding topographic maps are depicted in panel **(B)** for the contrast hits > cr in the time window 450–600 ms post-stimulus onset.

The analysis of ERPs to old and new items revealed that a frontal old/new effect reflecting familiarity was only significant in the control but not the patient group (see **Figure 2**). However, a significant parietal old/new effect, indicating recollection-based processing, was not found for either of the two groups. The contribution of familiarity and recollection was statistically assessed in an early and a late time window, respectively. Four anatomical ROIs were defined a priori to capture ERP effects at bilateral anterior and posterior scalp sites. Due to the expected spatial distribution of ERP old/new effects, the familiarity-based frontal old/new effect was analyzed in the early time window in left and right anterior ROIs and the parietal old/new effect reflecting the ERP correlate of recollection was assessed in the late time window in the left and right posterior ROIs. Statistics for old/new effects were computed in separate MANOVAs for the different ROIs.

#### Early time window

MANOVAs comprising the within-subjects factor Item Type (old, new) and the between-subjects factor Group (patients, controls) were computed separately for the left and right lateralized anterior ROIs. The respective analyses revealed no significant main effect of Item Type for the left ROI [*F*(1,22) = 1.32, *P* = 0.26] but a significant main effect of Item Type [*F*(1,22) = 9.74, *P* < 0.01] and a marginally significant two-way interaction of Item Type and Group [*F*(1,22) = 3.88, *P* = 0.06] for the right ROI. Because of strong a priori hypotheses concerning group differences between aMCI and control subjects, a follow-up analysis dissolving this interaction was performed for the right lateralized anterior ROI yielding a significant main effect of Item Type [*F*(1,9) = 7.17, *P* < 0.05, partial ŋ^2^ = 0.44] for the control group but no significant effect for the patient group [*F*(1,13) = 1.28, *P* = 0.28, partial ŋ^2^ = 0.09]. The associated ERP waveforms and topographic maps are illustrated in **Figure 2**.

#### Late time window

Corresponding MANOVAs containing the within-subject factor Item Type (old, new) and the between-subjects factor Group (patients, controls) did not show any significant effects of factor Item Type for the posterior ROIs [left: *F*(1,22) = 0.28, *P* = 0.6; right: *F*(1,22) = 2.07, *P* = 0.17] (see **Figure 2**).

### CORRELATION ANALYSES

The early frontal old/new effect was significantly positively correlated with performance in the recognition memory task (*r* = 0.44, *P* < 0.05) as well as the compound score of the memory tests (*r* = 0.47, *P* < 0.05). The recognition memory task was significantly positively correlated with the memory compound score (*r* = 0.64, *P* < 0.01). In contrast, the correlation of the frontal old/new effect with a compound score of executive functioning (comprising nine test scores, see **Table 1**) was not significant (*r* = 0.31, *P* = 0.15).

#### Structure-function correlations

The VBM regression analysis revealed a positive correlation of the GM volume of the left MTLC and the right fusiform gyrus (see **Table 4**) with a quantification of the ERP frontal old/new effect (mean amplitude of hits minus mean amplitude of correct rejections in the 450–600 ms time window at the anterior right hemisphere ROI; see **Figure 1B**), indicating that the ERP frontal old/new effect was larger the more GM volume was preserved in the inferior and medial temporal lobes. Besides the correlation with MTLC structures, also other neocortical areas in the medial temporal gyrus, lingual gyrus, dorsolateral prefrontal cortex and the insula are correlated with the familiarity ERP-effect.

## DISCUSSION

This study examined behavioral and electrophysiological correlates of recognition memory in single-domain aMCI patients with an assumed selective atrophy in the MTLC. Analyses of brain morphology by means of VBM confirmed a focused GM loss in the medial and inferior temporal lobes in the aMCI group compared to the healthy controls. The analysis of recognition memory performance showed poorer discrimination ability between correctly classified old and new items for the aMCI patients, which was accompanied by prolonged reaction times. Whereas a familiarity-related early frontally distributed ERP effect was present in the control group, no reliable frontal old/new effect could be found in the aMCI patient group. Additionally, neither group showed any sign of a significant parietal old/new effect indicating a lack of recollection-based processing. The magnitude of the frontal old/new effect correlated positively with test performance and with a compound measure including eight episodic memory test scores. Moreover, correlations of the frontal old/new effect with executive functioning comprised of an analogous compound score derived from neuropsychological testing turned out to be not significant. In addition, the size of the frontal old/new effect was positively correlated with the GM volume in the MTLC.

The behavioral results of this experiment are in line with our hypotheses that aMCI patients should perform more poorly than healthy seniors. The Petersen criteria define aMCI as a pathological condition with preserved general cognition but objectively impaired mnemonic functioning (Petersen, 2004; Winblad et al., 2004). In this study, this was ensured through a careful selection process where potential subjects’ cognitive functions were screened using multiple neuropsychological measures. The aMCI patient group differed significantly from the control group in four of eight episodic memory scores where they performed at least 1.3 SD inferior to the control group. Thus, also the behavioral data of the recognition memory paradigm showed a performance advantage for the healthy seniors.

The ERP results of the recognition memory experiment supported the hypotheses. Only the control group exhibited a significant frontal old/new effect. This corroborates recent findings of impaired familiarity in aMCI (e.g., Wolk et al., 2008; Algarabel et al., 2009). Contrary to these findings are the results of the behavioral studies by by Westerberg et al. (2006); Anderson et al. (2008), Hudon et al. (2009), and Serra et al. (2010) who reported familiarity to be preserved in aMCI patients. However, most of these studies examined recognition memory in mixed groups of multiple and single-domain aMCI patients with multiple-domain aMCI being the more frequent diagnosis. Thus, it is open to what extent these results are comparable to the current data that only incorporated subjects with a single-domain aMCI diagnosis, who are probably located at a transitional stage towards the development of AD. The outcome of patients suffering from mdMCI is less clear (Petersen, 2004; Fischer et al., 2007). The interpretation of the Anderson and Serra studies which only included single-domain aMCI patients is complicated because the Process Dissociation and the Remember/Know Procedure used in these studies have been called into question as being reliable behavioral measures of familiarity and recollection in healthy older subjects and in patients with aMCI due to intrinsic testing characteristics (e.g., Wolk et al., 2008; Serra et al., 2010). The Process Dissociation Procedure might lead to an underestimation of recognition memory performance in older adults because the exclusion condition requests to label the items from one of the two learning lists as new although they have been learned beforehand. Also, the Remember/Know procedure which assumes that the processes leading to one or the other introspectively determined judgment are independent might overestimate the impact of familiarity or recollection in subjects with specific memory problems.

The current results support the findings of Ally et al. (2009) who used a nearly identical paradigm with verbal and pictorial material in healthy older adults and aMCI patients. The current study only used verbal material and reveals an exact concordance with the Ally et al. (2009) data. They also found a significant frontal old/new effect only for the control subjects and no significant parietal old/new effect in both groups. Again, there is the question of precise comparability because Ally et al. (2009) also examined both single and multiple-domain aMCI patients. Thus the current results obtained in a more homogenous sample argue even more strongly in favor of impaired familiarity in very early developmental stages of AD caused by the neuropathological changes in the MTLC (Braak and Braak, 1991; Delacourte et al., 1999).

The scalp distribution of the familiarity effect in the control group was right-lateralized at frontal electrode sites. Usually the early old/new effect reflecting item familiarity is more mid-frontally focused (Rugg et al., 1998; Rugg and Curran, 2007). Friedman et al. (2010) compared the ERP correlates of familiarity and recollection across age groups and also found that older adults exhibited a rather right-lateralized frontal old/new effect whereas the analogous effect in adolescents and younger adults was mid-frontally focused. Identical results on topography were reported in a previous study investigating younger and older adults (Nessler et al., 2007). In both studies, it was hypothesized that the effect might have been lateralized at right hemisphere electrode sites due to additionally engaged monitoring processes to strengthen the processing of perceptual features to compensate for impaired recollection.

Neither of the groups showed a reliable old/new effect at parietal electrode sites. This indicates a lack of recollection-based processing. The study of Wolk et al. (2008) showed that MCI patients demonstrated not only familiarity but also recollection impairments relative to control subjects, in fact at least to the same extent. But here, also healthy seniors did not evoke a parietal recollection correlate. Friedman et al. (2010) clarified the developmental trajectories of familiarity and recollection in a lifespan perspective but only healthy seniors were included in their sample. A parietal old/new effect was indeed present across age groups in that study but the magnitude of the effect diminished with increasing age. Moreover, their subjects had to learn visual abstract material over four study-test cycles leading to a more pronounced recollection effect in three younger age groups with repetition but not in the senior group. Hence, the results of Friedman et al. (2010), suggest that the distorted recollection ERP correlates in old age rely on impaired recollection. An age-related decline in recollection performance is also corroborated by behavioral and imaging studies (e.g., Parkin and Walter, 1992; Cabeza et al., 2004). Recollection relies on the integrity of the hippocampus (e.g., Eldridge et al., 2000; Baddeley et al., 2001; Eichenbaum et al., 2007) and aging studies showed that especially hippocampal tissue is vulnerable to age-related shrinkage (e.g., Raz et al., 2005). Thus, the absence of an ERP correlate of recollection in healthy seniors can be accounted for by the natural decline going along with aging. Another account for the absence of a recollection correlate might be inherent in the nature of the task. Subjects had to learn a list of single words and were subsequently tested to discriminate between previously seen old words and new words. Because there was neither a subsequent query for associational information nor a source judgment, it was not necessary for the subjects to recall any further details from the study episode besides the targeted word forms to successfully accomplish this task. Thus, it might have been irrelevant for the task to engage recollective processing to retrieve any specific details. Potentially, some but not all of the participants might have initiated recollection processes, leading to greater inter-subject variability (Sugiura et al., 2007). This might have resulted in a reduction of an ERP correlate of recollection.

The significant positive correlations of the behavioral assessments of episodic memory capacity and the magnitude of the familiarity-related ERP correlate suggest that the decline in memory performance covaries with a decrease of the frontal old/new effect.

The supplementary examination of the GM volume of MTL structures revealed bilateral shrinkage of aMCI patients’ GM volume within the medial and inferior temporal lobe, specifically within the perirhinal cortex and the fusiform gyrus. Moreover, GM volume of MTLC structures covaried positively with the magnitude of the early frontal ERP effect. To our knowledge these are the first data which can show a direct link between the MTLC and the putative electrophysiological correlate of familiarity.

Structural alterations in medial and inferior temporal lobe have been reported in several studies investigating morphometric changes in aMCI patients, whereby volume loss in rhinal cortex and in fusiform gyrus was repeatedly found (Convit et al., 2000; Karas et al., 2004; Chételat et al., 2005; Bozzali et al., 2006; Barbeau et al., 2008; Schmidt-Wilcke et al., 2009). Moreover, in aMCI patients MTLC volumes have also been found to be positively correlated with performance in mnemonic neuropsychological tests (Meyer et al., 2013). Neither a volumetric between-group difference in hippocampal GM nor a correlation of hippocampal GM and the ERP effect could be found. Thus in the case of patients at a very early stage of presumed AD pathology, the examination of MTLC atrophy might be more useful to support an early diagnosis of AD-related neuropathology than hippocampal atrophy. On the other hand, volume loss of hippocampal GM that exceeds atrophic changes associated with healthy aging might be suited to differentiate between healthy elderly and patients only at a later stage of the neuropathological process. In addition, the present data suggest that alteration of the electrophysiological correlate of familiarity might be a suitable marker of these early subtle cognitive and anatomical changes.

Concerning the correlations of familiarity with GM volume outside the MTLC, several fMRI studies also reported activations in the medial temporal gyrus (Henson et al., 1999; Yonelinas et al., 2001; Montaldi et al., 2006), the lingual gyrus (Voss et al., 2008), the insula (Henson et al., 1999; Montaldi et al., 2006) and the dorsolateteral prefrontal cortex (Yonelinas et al., 2005) which were correlated with familiarity strength. Thus, it is well conceivable that differences in GM in these regions also contribute to a modulation of the familiarity signal.

One major critical point when examining MCI patients is who of the aMCI subjects will prospectively develop AD. This question can only be answered when longitudinal studies are conducted. There have been attempts to find predictors of subsequent conversion including volumetric measures of hippocampal and entorhinal volumes (e.g., Pennanen et al., 2004). The results of the current study suggest that the deficiency of an ERP correlate of familiarity, or at least the attenuation of such an ERP effect, might be a supplementary predictor of conversion to AD.

One potential limitation of this study is the rather small sample size of only fourteen aMCI patients and ten healthy controls. Whereas for the ERP analysis, the reported effects were statistically significant and seemed rather robust (in terms of effect sizes), for the VBM analysis, only effects with p-levels uncorrected for multiple comparisons could be reported. This might actually be due to a lack of power because of the small sample size. A second limitation might be the significantly poorer performance of the aMCI subjects in the recognition memory task. It could be a possible confound that the two groups differ in their performance levels but because the defined clinical criteria for a diagnosis of aMCI include an objective impairment in memory tests, it is not surprising that the patients yielded poorer Pr scores in the experimental task. But it is important to state that also the patient group did not operate at chance level in the recognition task (a Pr score of 0.74 is significantly different from 0.5). Finally, the current results do not allow to draw strong conclusions about the location of the neural generators of the familiarity-related early old/new effect as scalp ERPs cannot be used to determine the neural generators with high precision. However, the current electrophysiological and correlational results clearly support the notion that the familiarity-related early old/new effect relies upon the integrity of the MTLC.

## CONCLUSION

The current data provide evidence that aMCI patients exhibit a specific memory-related cognitive impairment. The electrophysiological correlate of familiarity substantially differs between aMCI patients and age-matched healthy controls. Those alterations can be ascribed to neuropathological changes in the MTLC. Thus, the detection of abnormal ERP correlates of familiarity, together with a neuropsychologically based diagnosis of aMCI, might constitute an important predictor of an underlying AD pathology and if validated in further studies, may be used as a biomarker for AD.

## Conflict of Interest Statement

The authors declare that the research was conducted in the absence of any commercial or financial relationships that could be construed as a potential conflict of interest.

## Footnotes

5

6

7

## References

[B1] Addante R. J. Ranganath C. Olichney J. Yonelinas A. P. (2012). Neurophysiological evidence for a recollection impairment in amnesia patients that leaves familiarity intact. *Neuropsychologia* 50 3004–3014 10.1016/j.neuropsychologia.2012.07.038 22898646 PMC3483383

[B2] Aggleton J. P. Brown M. W. (1999). Episodic memory, amnesia, and the hippocampal-anterior thalamic axis. *Behav. Brain. Sci.* 22 425–444 10.1017/S0140525X99002034 11301518

[B3] Algarabel S. Escudero J. Mazón J. F. Pitarque A. Fuentes M. Peset V. (2009). Familiarity-based recognition in the young, healthy elderly, mild cognitive impairment and Alzheimer’s patients. *Neuropsychologia* 47 2056–2064 10.1016/j.neuropsychologia.2009.03.016 19467356

[B4] Algarabel S. Fuentes M. Escudero J. Pitarque A. Peset V. Mazón J. F. (2012). Recognition memory deficits in mild cognitive impairment. *Aging Neuropsychol. Cogn.* 19 608–619 10.1080/13825585.2011.640657 22247955

[B5] Ally B. A. McKeever J. D. Waring J. D. Budson A. E. (2009). Preserved frontal memorial processing for pictures in patients with mild cognitive impairment. *Neuropsychologia* 47 2044–2055 10.1016/j.neuropsychologia.2009.03.015 19467355 PMC2724267

[B6] Anderson N. D. Ebert P. L. Jennings J. M. Grady C. L. Cabeza R. Graham S. J. (2008). Recollection- and familiarity-based memory in healthy aging and amnestic mild cognitive impairment. *Neuropsychology* 22 177–187 10.1037/0894-4105.22.2.177 18331160

[B7] Ashburner J. (2007). A fast diffeomorphic image registration algorithm. *Neuroimage* 38 95–113 10.1016/j.neuroimage.2007.07.007 17761438

[B8] Baayen H. Piepenbrock R van Rijn H. (1993). *The CELEX lexical database (CD-ROM)*. Philadelphia: Linguistic Data Consortium, University of Pennsylvania

[B9] Baddeley A. Vargha-Khadem F. Mishkin M. (2001). Preserved recognition in a case of developmental amnesia: implications for the acquisition of semantic memory? *J. Cogn. Neurosci.* 13 357–369 10.1162/08989290151137403 11371313

[B10] Barbeau E. J. Ranjeva J. P. Didic M. Confort-Gouny S. Felician O. Soulier E. (2008). Profile of memory impairment and gray matter loss in amnestic mild cognitive impairment. *Neuropsychologia* 46 1009–1019 10.1016/j.neuropsychologia.2007.11.019 18191160

[B11] Bell-McGinty S. Lopez O. L. Cidis Meltzer C. Scanlon J. M. Whyte E. M. DeKosky S. T. (2005). Differential cortical atrophy in subgroups of mild cognitive impairment. *Arch. Neurol.* 62 1393–1397 10.1001/archneur.62.9.1393 16157746

[B12] Bowles B. Crupi C. Mirsattari S. M. Pigott S. E. Parrent A. G. Pruessner J. C. (2007). Impaired familiarity with preserved recollection after anterior temporal-lobe resection that spares the hippocampus. *Proc. Natl. Acad. Sci. U.S.A.* 104 16382–16387 10.1073/pnas.0705273104 17905870 PMC1995093

[B13] Bozzali M. Filippi M. Magnani G. Cercignani M. Franceschi M. Schiatti E. (2006). The contribution of voxel-based morphometry in staging patients with mild cognitive impairment. *Neurology* 67 453–460 10.1212/01.wnl.0000228243.56665.c2 16894107

[B14] Braak H. Braak E. (1991). Neuropathological stageing of Alzheimer-related changes. *Acta Neuropathol.* 82 239–259 10.1007/BF00308809 1759558

[B15] Brandt J. Spencer M. Folstein M. (1988). The telephone interview for cognitive status. *Neuropsychiatry Neuropsychol. Behav. Neurol.* 1 111–117

[B16] Busatto G. F. Diniz B. S. Zanetti M. V. (2008). Voxel-based morphometry in Alzheimer’s disease. *Expert Rev. Neurother.* 8 1691–1702 10.1586/14737175.8.11.1691 18986240

[B17] Cabeza R. Daselaar S. M. Dolcos F. Prince S. E. Budde M. Nyberg L. (2004). Task-independent and task-specific age effects on brain activity during working memory, visual attention and episodic retrieval. *Cereb. Cortex* 14 364–375 10.1093/cercor/bhg133 15028641

[B18] Chételat G. Landeau B. Eustache F. Mézenge F. Viader F. de la Sayette V. (2005). Using voxel-based morphometry to map the structural changes associated with rapid conversion in MCI: a longitudinal MRI study. *Neuroimage * 27 934–946 10.1016/j.neuroimage.2005.05.015 15979341

[B19] Convit A. de Asisc J. de Leon M. J. Tarshish C. Y. De Santi S. Rusinek H. (2000). Atrophy of the medial occipitotemporal, inferior, and middle temporal gyri in non-demented elderly predict decline to Alzheimer’s disease. *Neurobiol. Aging* 21 19–26 10.1016/S0197-4580(99)00107-104 10794844

[B20] Delacourte A. David J. P. Sergeant N. Buée L. Wattez A. Vermersch P. (1999). The biochemical pathway of neurofibrillary degeneration in aging and Alzheimer’s disease. *Neurology* 52 1158–1165 10.1212/WNL.52.6.1158 10214737

[B21] Dennis N. A. Cabeza R. (2008). “Neuroimaging of healthy cognitive aging,” in *The Handbook of Aging and Cognition* 3rd Edn eds Craik F. I. M. Salthouse T. A. (New York: Psychology Press) 1–54

[B22] Dien J. Santuzzi A. M. (2005). “Application of repeated measures ANOVA to high-density ERP datasets: a review and tutorial,” in *Event-Related Potentials: A Methods Handbook* ed. Handy T. C. (Cambridge: MIT Press) 57–82

[B23] Duarte A. Ranganath C. Trujillo C. Knight R. T. (2006). Intact recollection memory in high-performing older adults: ERP and behavioural evidence. *J. Cogn. Neurosci.* 18 33–47 10.1162/089892906775249988 16417681

[B24] Dubois B. Feldman H. H. Jacova C. Cummings J. L. DeKosky S. T. Barberger-Gateau P. (2010). Revising the definition of Alzheimer’s disease: a new lexicon. *Lancet Neurol.* 9 1118–1127 10.1016/S1474-4422(10)70223-70224 20934914

[B25] Düzel E. Habib R. Schott B. Schoenfeld A. Lobaugh N. McIntosh A. R. (2003). A multivariate, spatiotemporal analysis of electromagnetic time-frequency data of recognition memory. *Neuroimage* 18 185–197 10.1016/S1053-8119(02)0031-39 12595175

[B26] Düzel E. Vargha-Khadem F. Heinze H.-J. Mishkin M. (2001). Brain activity evidence for recognition without recollection after early hippocampal damage. *Proc. Natl. Acad. Sci. U.S.A.* 98 8101–8106 10.1073/pnas.131205798 11438748 PMC35474

[B27] Eichenbaum H. Yonelinas A. P. Ranganath C. (2007). The medial temporal lobe and recognition memory. *Annu. Rev. Neurosci.* 30 123–152 10.1146/annurev.neuro.30.051606.094328 17417939 PMC2064941

[B28] Eldridge L. L. Knowlton B. J. Furmanski C. S. Bookheimer S. Y. Engel S. A. (2000). Remembering episodes: a selective role for the hippocampus during retrieval. *Nat. Neurosci.* 3 1149–1152 10.1038/80671 11036273

[B29] Fernandez G. Tendolkar I. (2006). The rhinal cortex: ‘gatekeeper’ of the declarative memory system. *Trends Cogn. Sci.* 10 358–362 10.1016/j.tics.2006.06.003 16843039

[B30] First M. B. Spitzer R. L. Gibbon M Williams J. B. W. (2002). *Structured Clinical Interview for DSM-IV-TR Axis I Disorders, Research Version, Nonpatient Edition SCID-I/NP*. New York: Biometrics Research, New York State Psychiatric Institute

[B31] Fischer P. Jungwirth S. Zehetmayer S. Weissgram S. Hoenigschnabl S. Gelpi E. (2007). Conversion from subtypes of mild cognitive impairment to Alzheimer dementia. *Neurology* 68 288–291 10.1212/01.wnl.0000252358.03285.9d 17242334

[B32] Friedman D. de Chastelaine M. Nessler D. Malcolm B. (2010). Changes in familiarity and recollection across the lifespan: an ERP perspective. *Brain Res.* 1310 124–141 10.1016/j.brainres.2009.11.016 19914220 PMC2812671

[B33] Gauggel S. Deckersbach T. Rolko C. (1998). Development and evaluation of a rating scale to assess problem-solving and planning deficits. *Zeitschrift fü*r Neuropsychologie 9 3–17

[B34] Gonsalves B. D. Kahn I. Curran T. Norman K. A. Wagner A. D. (2005). Memory strength and repetition suppression: multimodal imaging of medial temporal cortical contributions to recognition. *Neuron* 47 751–761 10.1016/j.neuron.2005.07.013 16129403

[B35] Good C. D. Johnsrude I. S. Ashburner J. Henson R. N. A. Friston K. J Frackowiak R. S. J. (2001). A voxel-based morphometric study of ageing in 465 normal adult human brains. *Neuroimage* 14 21–36 10.1006/nimg.2001.0786 11525331

[B36] Grunwald T. Lehnertz K. Heinze H. J. Helmstaedter C. Elger C. E. (1998). Verbal novelty detection within the human hippocampus proper. *Proc. Natl. Acad. Sci. U.S.A.* 95 3193–3197 10.1073/pnas.95.6.3193 9501239 PMC19718

[B37] Henson R. N. A. Rugg M. D. Shallice T. Josephs O. Dolan R. J. (1999). Recollection and familiarity in recognition memory: an event-related functional magnetic resonance imaging study. *J. Neurosci.* 19 3962–3972 10234026 10.1523/JNEUROSCI.19-10-03962.1999 PMC6782715

[B38] Hudon C. Belleville S. Gauthier S. (2009). The assessment of recognition memory using the remember/know procedure in amnestic mild cognitive impairment and probable Alzheimer’s disease. *Brain Cogn.* 70 171–179 10.1016/j.bandc.2009.01.009 19250730

[B39] Karas G. B. Scheltens P. Rombouts S. A. R.B. Visser P. J. van Schijndel R. A. Fox N. C. (2004). Global and local gray matter loss in mild cognitive impairment and Alzheimer’s disease. *Neuroimage* 23 708–716 10.1016/j.neuroimage.2004.07.006 15488420

[B40] Kordower J. H. Chu Y. Stebbins G. T. DeKosky S. T. Cochran E. J. Bennett D. (2001). Loss and atrophy of layer II entorhinal cortex neurons in elderly people with mild cognitive impairment. *Ann. Neurol.* 49 202–213 10.1002/1531-8249(20010201)49:2<202::AID-ANA40>3.0.CO;2-3 11220740

[B41] Meyer P. Feldkamp H. Hoppstädter M. King A. V. Frölich L. Wessa M. (2013). Using voxel-based morphometry to examine the relationship between regional brain volumes and memory performance in amnestic mild cognitive impairment. *Front. Behav. Neurosci. *7:89. 10.3389/fnbeh.2013.00089 PMC3719379 23888131

[B42] Montaldi D. Spencer T. J. Roberts N. Mayes A. R. (2006). The neural system that mediates familiarity memory. *Hippocampus* 16 504–520 10.1002/hipo.20178 16634088

[B43] Morris J. C. Heyman A. Mohs R. C. Hughes J. P. van Belle G. Fillenbaum G. (1989). The Consortium to Establish a Registry for Alzheimer’s Disease (CERAD). Part I. clinical and neuropsychological assessment of Alzheimer’s disease*. Neurology* 39 1159–1165 10.1212/WNL.39.9.1159 2771064

[B44] Nessler D. Friedman D. Johnson R. Bersick M. (2007). Does repetition engender the same retrieval processes in young and older adults? *Neuroreport* 18 1837–1840 10.1097/WNR.0b013e3282f16d9f 18090322

[B45] Parkin A. J. Walter B. M. (1992). Recollective experience, normal aging, and frontal dysfunction. *Psychol. Aging* 7 290–298 10.1037//0882-7974.7.2.290 1610518

[B46] Pennanen C. Kivipelto M. Tuomainen S. Hartikainen P. Hänninen T. Laakso M. P. (2004). Hippocampus and entorhinal cortex in mild cognitive impairment and early AD. *Neurobiol. Aging* 25 303–310 10.1016/S0197-4580(03)00084-88 15123335

[B47] Petersen R. C. (2004). Mild cognitive impairment as a diagnostic entity. *J. Intern. Med.* 256 183–194 10.1111/j.1365-2796.2004.01388.x 15324362

[B48] Petersen R. C. Smith G. E. Waring S. C. Ivnik R. J. Tangalos E. G. Kokmen E. (1999). Mild cognitive impairment: clinical characterization and outcome. *Arch. Neurol.* 56 303–308 10.1001/archneur.56.3.303 10190820

[B49] Pihlajamäki M. Jauhiainen A. M. Soininen H. (2009). Structural and functional MRI in mild cognitive impairment. *Curr. Alzheimer Res.* 6 179–185 10.2174/156720509787602898 19355853

[B50] Raz N. Lindenberger U. Rodrigue K. M. Kennedy K. M. Head D. Williamson A. (2005). Regional brain changes in aging healthy adults: general trends, individual differences and modifiers. *Cereb. Cortex* 15 1676–1689 10.1093/cercor/bhi044 15703252

[B51] Rugg M. D. Curran T. (2007). Event-related potentials and recognition memory. *Trends Cogn. Sci.* 11 251–257 10.1016/j.tics.2007.04.004 17481940

[B52] Rugg M. D. Mark R. E. Walla P. Schloerscheidt A. M. Birch C. S. Allan K. (1998). Dissociation of the neural correlates of implicit and explicit memory. *Nature* 392 595–598 10.1038/33396 9560154

[B53] Schmidt-Wilcke T. Poljansky S. Hierlmeier S. Hausner J. Ibach B. (2009). Memory performance correlates with gray matter density in the ento-/perirhinal cortex and posterior hippocampus in patients with mild cognitive impairment and healthy controls – a voxel based morphometry study. *Neuroimage* 47 1914–1920 10.1016/j.neuroimage.2009.04.092 19442751

[B54] Serra L. Bozzali M. Cercignani M. Perri R. Fadda L. Caltagirone C. (2010). Recollection and familiarity in amnesic mild cognitive impairment. *Neuropsychology* 24 316–326 10.1037/a0017654 20438209

[B55] Snodgrass J. G. Corwin J. (1988). Pragmatics of measuring recognition memory: applications to dementia and amnesia. *J. Exp. Psychol. Gen.* 117 34–50 10.1037/0096-3445.117.1.34 2966230

[B56] Sperling R. A. Aisen P. S. Beckett L. A. Bennett D. A. Craft S. Fagan A. M. (2011). Toward defining the preclinical stages of Alzheimer’s disease: recommendations from the National Institute on Aging and the Alzheimer’s Association workgroup. *Alzheimers Dement.* 7 280–292 10.1016/j.jalz.2011.03.003 21514248 PMC3220946

[B57] Sugiura M. Friston K. J. Willmes K. Shah N. J. Zilles K. Fink G. R. (2007). Analysis of intersubject variability in activation: an application to the incidental episodic retrieval during recognition test. *Hum. Brain Mapp.* 28 49–58 10.1002/hbm.20256 16718653 PMC6871327

[B58] Voss J. L. Reber P. J. Mesulam M. M. Parrish T. B. Paller K. A. (2008). Familiarity and conceptual priming engage distinct cortical networks. *Cereb. Cortex* 18 1712–1719 10.1093/cercor/bhm200 18056085 PMC2736907

[B59] Wang T. H. de Castelaine M. Minton B. Rugg M. D. (2011). Effects of age on the neural correlates of familarity as indexed by ERPs. *J. Cogn. Neurosci.* 23 1–14 10.1162/jocn_a_00129 21878056 PMC3262081

[B60] Wechsler D. (1987). *Wechsler Memory Scale-Revised*. New York: Harcourt Brace Jovanovich

[B61] Westerberg C. E. Paller K. A. Weintraub S. Mesulam M.-M. Holdstock J. S. Mayes A. R. (2006). When memory does not fail: familiarity-based recognition in mild cognitive impairment and Alzheimer’s disease. *Neuropsychology* 20 193–205 10.1037/0894-4105.20.2.193 16594780

[B62] Whitwell J. L. Petersen R. C. Negash S. Weigand S. D. Kantarci K. Ivnik R. J. (2007). Patterns of atrophy differ among specific subtypes of mild cognitive impairment. *Arch. Neurol.* 64 1130–1138 10.1001/archneur.64.8.1130 17698703 PMC2735186

[B63] Winblad B. Palmer K. Kivipelto M. Jelic V. Fratiglioni L. Wahlund L.-O. (2004). Mild cognitive impairment – beyond controversies, towards a consensus: report of the international working group on mild cognitive impairment. *J. Intern. Med.* 256 240–246 10.1111/j.1365-2796.2004.01380.x 15324367

[B64] Wolk D. A. Dickerson B. C. (2011). Fractionating verbal episodic memory in Alzheimer’s Disease. *Neuroimage* 54 1530–1539 10.1016/j.neuroimage.2010.09.005 20832485 PMC2997155

[B65] Wolk D. A. Dunfee K. L. Dickerson B. C. Aizenstein H. J. DeKosky S. T. (2011). A medial temporal lobe division of labor: insights from memory in aging and early Alzheimer disease. *Hippocampus* 21 461–466 10.1002/hipo.20779 20232383 PMC2918673

[B66] Wolk D. A. Signoff E. D. DeKosky S. T. (2008). Recollection and familiarity in amnestic mild cognitive impairment: a global decline in recognition memory. *Neuropsychologia* 46 1965–1978 10.1016/j.neuropsychologia.2008.01.017 18328509 PMC2519866

[B67] Yonelinas A. P. (2002). The nature of recollection and familiarity: a review of 30 years of research. *J. Mem. Lang.* 46 441–517 10.1006/jmla.2002.2864

[B68] Yonelinas A. P. Hopfinger J. B. Buonocore M. H. Kroll N. E. A. Baynes K. (2001). Hippocampal, parahippocampal and occipital-temporal contributions to associative and item recognition memory: an fMRI study. *Neuroreport* 12 359–363 10.1097/00001756-200102120-00035 11209950

[B69] Yonelinas A. P. Otten L. J. Shaw K. N. Rugg M. D. (2005). Separating the brain regions involved in recollection and familiarity in recognition memory. *J. Neurosci.* 25 3002–3008 10.1523/JNEUROSCI.5295-04.2005 15772360 PMC6725129

